# A text mining analysis of human flourishing on Twitter

**DOI:** 10.1038/s41598-023-30209-7

**Published:** 2023-02-28

**Authors:** Manuel Cebral-Loureda, Alberto Hernández-Baqueiro, Enrique Tamés-Muñoz

**Affiliations:** 1 Department of Humanistic Studies, Tecnologico de Monterrey, 64849 Monterrey, Mexico; 2 Department of Humanistic Studies, Tecnologico de Monterrey, 14380 Mexico, Mexico; 3 Human Flourishing Projects, Tecnologico de Monterrey, 64849 Monterrey, Mexico

**Keywords:** Psychology, Human behaviour, Computational science

## Abstract

The power of social media in spreading the idea of wellbeing has already been addressed by several psychologists and scholars through the analysis of the vocabulary; however, the use of the human flourishing (HF) concept in such platforms has not yet been analyzed. This study addresses such a topic by analyzing more than 600 thousand Twitter messages posted by a community of users who associate themselves with HF and comparing them to more than 400 thousand messages in other Twitter lists. The study aims to identify the HF users’ interests, the richness in their vocabulary, the feelings and emotions that they share, and the grammar used in their constructions. Such an analysis was conducted through text mining computational methods, including sentiment analysis, natural language processing (NLP), and topic modeling. The results revealed that although HF users employ average vocabulary diversity, they share more positive emotions, and a greater variety of emojis. They also tended to discuss different topics, from more spiritual and health-related subjects to more practical matters related to work and success. Finally, they generally wrote from an empathetic state of mind, caring about people’s day-to-day feelings and about the world.

## Introduction

The idea that changing language changes perception has already been addressed by wellbeing studies. Seligman referred to the power of social media in spreading the idea of wellbeing, and how it could be studied by analyzing the vocabulary used in posts^1^. Just two years later, a study conducted by^2^ explored the use of language in Facebook, finding similarities and differences between groups defined by known demographic and psychological characteristics. Recently, the Center for Wellbeing Science at the University of Melbourne has been promoting a Wellbeing Literacy model (WBL) for scientific research and practice, seeking to understand how wellbeing messages are written in diverse contexts to maintain or improve both personal wellbeing and that of others and the world^3^.

In general, more studies can be found which analyze the relationship between language richness and wellbeing^4–9^; however, none of them focus on Human Flourishing (HF), a much more metaphorical term defined as the optimal and continuous development of human potentialities, including their relationships and participation in meaningful activities^10^. Because of the variety of understandings that human beings may have of what is meaningful, it is useful to know how the concept of HF is socially constructed. To address this topic, this study analyzed the messages posted on the Twitter social network by a community of users who associated themselves with HF. The research aims to identify this community of users’ interests, their vocabulary richness, the feelings and emotions that they shared, and their grammar constructions.

## Materials and methods

To analyze the production of Twitter messages addressing HF, it was necessary to identify the population immersed in this subject, so the term *human flourishing* was searched across Twitter, finding a total of 47 users who explicitly affirmed to be associated with HF. However, while many other users did not mention both terms -human flourishing- in their profile definition, they did use the verb *flourish* to refer to a human condition or activity; for example, they defined themselves as flourishing mothers or fathers; there were flourishing food, restaurants, and therapies; also, writers or coaches for flourishing; and even jobs and businesses for flourishing. Undoubtedly these users are alluding to a process of human and/or social flourishing, so they were added to those identified above, reaching a total of 442 users, with which a list of users on Twitter was created.

With such a Twitter list created, it was easier to access all these users' timelines by means of the Twitter API, included in the *rtweet* package^11^ through the function *get_timelines*. Although the function has a limit of 3200 tweets for retrieval per user, it is possible to perform recurrent requests to create an extensive database. After removing those tweets which might be duplicated because of the recursivity of the operation, a total of 670,000 tweets belonging to the identified HF users’ timelines were collected.

In addition to this HF list, other lists were inspected for a comparative framework of reference. Five very trending lists were chosen, following the selection of a digital market blog^12^, namely the list of Top Twitter Influencers; the VC A-List about venture folk; the Journalism list; a Startup A-List; and a Business Finance list (Table 1). As with the HF list, their tweets were collected through the *rtweet* package, although in these cases, the recurrent retrieval was not applied since these lists were used only for metrical comparison and not to submit them to a later study of their specific vocabulary.

**Table 1:** Summary of the data collected from the HF Twitter list as well as other five lists for comparison.

Human flourishing research	Top Twitter influencers	VC A-list
List for a Human Flourishing Social Media Research|437 Members|9 Followers **670 K tweets collected**	As ranked by Forbes and admired by us|46 Members|1.7 K Followers **45 K tweets collected**	Following top venture folk worldwide|104 Members|735 Followers **93 K tweets collected**

Journalism	Startup A-list	Business finance
Websites and people that journalism students should know|106 Members|534 Followers **100 K tweets collected**	Following top startup accounts worldwide|168 Members|913 Followers **151 K tweets collected**	Experts in Business Finance who are most followed by other experts|237 Members|352 Followers **227 K tweets collected**

Besides the difference in the number of tweets collected, there is also a significant difference in the number of followers of each list. This is because the lists selected for comparison are included among the most trending in Twitter; while the HF list was created solely for the purposes of this research. Furthermore, the high level of followers in the comparison lists is considered a value for the study, since the HF list is being compared with very active and influential users.

### Text mining techniques

In order to explore the content of the messages, the texts in the collected tweets were analyzed using text mining techniques. Firstly, the content of the tweets was tokenized using the *tidytext* package^13^ obtaining the most frequent words for each of the collected lists. Typically, if the vocabulary of a language is ranked by frequency of use, it follows the so-called Zipf’s law^14^, which states that the frequency of any word is inversely proportional to its rank in the frequency table. However, slight nuances can appear when the rank-frequency relation plummets, thus indicating a more concentrated use of certain words and, therefore, a less distributed vocabulary use. Of course, this is a highly specific quantitative approach that should be complemented in order to better understand the vocabulary richness of a text^15^. This was achieved by applying a variation of Zipf's law, the so-called Heaps’ law, which measures the growth in the number of distinct words in a document in relation to the document’s length^16^. The two methods, along with the vocabulary density of each list (the number of distinct words divided by the total number of words) offered a more accurate idea of the word distribution in each analyzed list.

A second approximation undertaken was emotion recognition, using the previous tokenization together with the NRC emotion lexicon^17,18^. This lexicon gives to the predetermined 14,182-word repository an emotional value by categorizing it into eight main emotions: trust, joy, anticipation, surprise, fear, disgust, sadness, and anger. For each emotion, each word in the NRC repository receives a value of either one or zero, depending on whether the term is considered to evoke such emotion or not. By counting the times that a word in the NRC repository appears in a text, a resulting emotional value is calculated. The NRC lexicon also classifies the same words by giving them either a positive or negative sentiment value. The emotion recognition and the sentiment analysis developed through the NRC repository are part of the so-called Lexicon-Based Methods, which have proven to be robust, less domain-dependent than other text classification methods, and just as useful for social media analysis^19^.

Furthermore, taking into account that emojis play an essential role in the construction of sentence meaning, especially within the social media context^20,21^, they were extracted using the *emoji_extract* function of the *emoji* package^22^ and their frequency was counted and represented as if they were normal words. Together with other slang words, emojis express a kind of crowd wisdom^23^ which is quite helpful in understanding social exchanges among user sets such as those of HF.

### NLP methods

A more sophisticated technique used was entity recognition, a natural language processing (NLP) technique. It was applied by using the *udpipe* package^24^, which enables the use of pre-trained models. Among them, the *UD_English-EWT* model^25^ proved to be accurate for the study’s purpose, since it comprises 254,825 words and 16,621 sentences taken from weblogs, newsgroups, emails, reviews, and Yahoo! answers. In keeping with an NLP model, the tool processes a given corpus word by word and sentence by sentence, retrieving an annotated corpus and showing variables such as lemmas, the roots of words as they are recorded in a dictionary; POS tags, taggings that label words by their part of the speech; and features, the temporal forms of nouns and verbs. As other studies have proven before, these methods are useful for a better understanding of sentiment and mood on social media^26,27^.

### LDA for topic modeling

Finally, to gain a deeper insight on the content of the tweets, the study used topic modeling text-classification technique. This technique is usually applied through the Latent Dirichlet allocation (LDA) algorithm, available in the *topicmodels* package^28^ by means of the *LDA* function. This algorithm classifies every document as a set of topics and each topic as a set of terms^29^. With these references, the LDA algorithm creates an unsupervised vector space in which it lists the topics according to the vectors that relate terms to the documents. As a result, LDA offers two values: *beta*, which expresses the strength of each term’s connection with each topic; and *gamma*, which provides the strength of the document's connection to the topics. This study analyzed the words in the HF list, once the stopwords had been removed; on the other hand, the whole production of each user's timeline was considered a document.

Another of LDA's configurable values is k, which indicates the number of topics that LDA will search. This value has been established by a previous study to calculate an efficient number of topics for a dataset. This constitutes the perplexity measure^29^, also available in the *topicmodels* package through the *perplexity* function^28^. In this work, the perplexity values for the HF sample were 5443.457, 5127.896, 4669.519, 4475.305, 4409.658, and 4265.236 for k equal to 2, 3, 4, 5, 6, and 7 respectively. Although topic division is better when the perplexity measure is lower, when using greater values for k, it is normal for the perplexity value to decrease. The objective is, thus, to find a k value that implies a significant decrease with respect to its precedent one and a small drop regarding the following one. Four (4) was the value chosen from the obtained results, so four final topics were generated for the tweets in the HF list.

### Visualizations

In order to synthesize the graphical representations and facilitate the comparison, many of the plots were deployed together by means of the *ggarrange* function of the *ggpubr* package^30^.

## Findings

In a first visual scan, it was observed that -as is typical for a social networking platform and even more so when it comes to HF- many user profile pictures showed a profile picture of the person, usually smiling amicably. In fact, such photos made up half of the images (Table 2). However, there were also many other profiles represented by a logo, most of them with a white background and green text. This initial overview alone clearly shows that HF does not only refer to individuals, but that many companies or projects also use the concept to describe themselves on Twitter.

**Table 2:** Overview of the type of profiles included in the HF Twitter list observing their photo profile.

Type of photo profile	Number of occurrences	Percentage
Portrait personal photo	209	47%
Logo	200	45%
Other (pictures, drawings, etc.)	26	6%
No photo profile	7	2%

### Zipf’s law, heaps’ law, and density comparison

**Figure 1:**
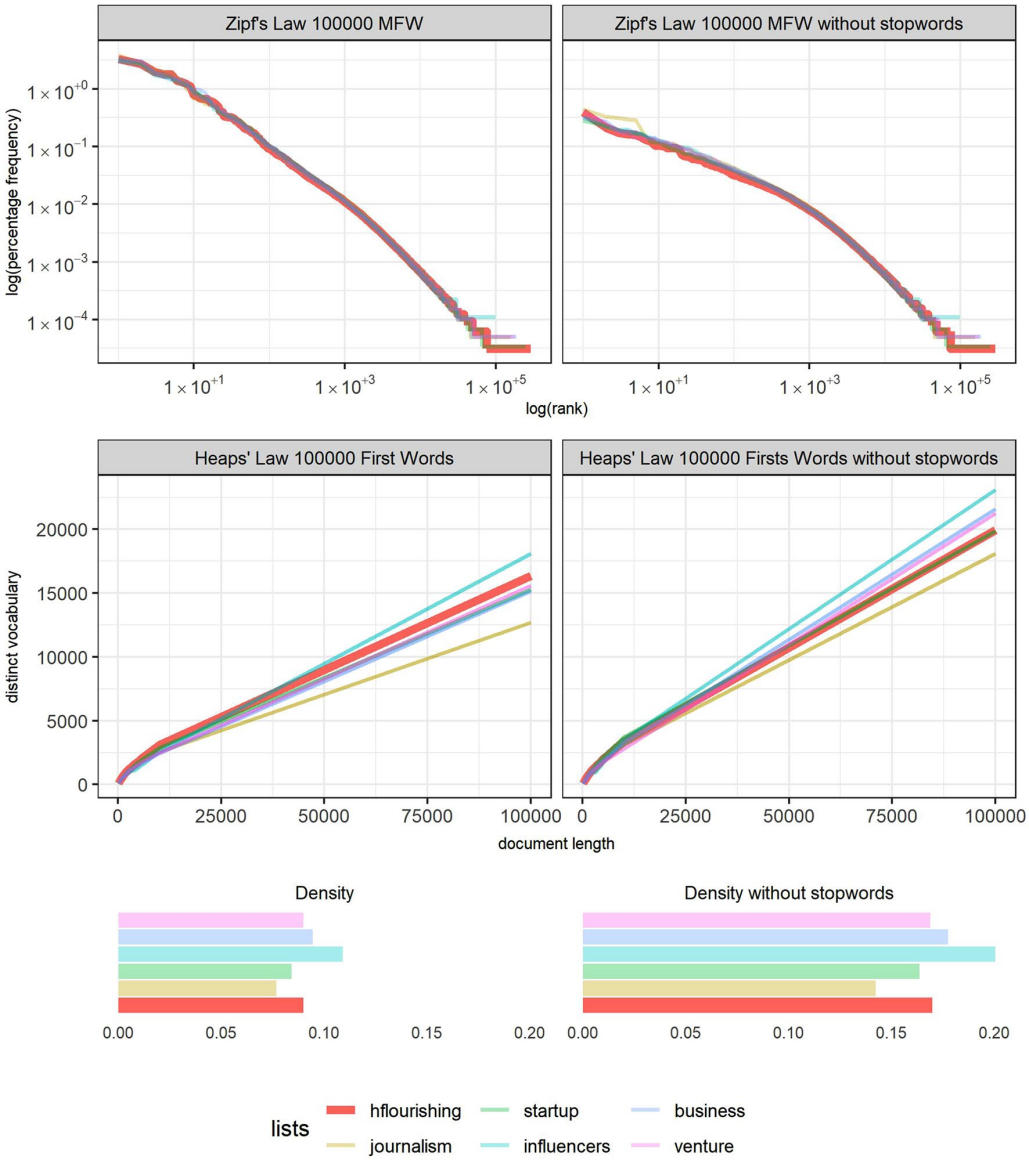
Plots displaying the Zipf’s Law, Heaps’ Law, and density statistics measures. Colors show each of the lists, with the HF list highlighted by harder and wider strokes. The plots were made by the authors, using R programming.

Already properly applying the techniques of text mining, the content of the tweets was analyzed, comparing the HF list with the other selected lists. First, a check for Zipf's Law in the vocabulary produced by each list revealed that they all tended to occupy the inverse diagonal of the plot but with slight differences. Specifically, when all the 50,000 most frequent words (MFW) were considered, the vocabulary of the HF list decreased slightly faster than the others (upper left part in Fig. 1). This situation became accentuated after removing the stopwords (upper right part in Fig. 1). Between ranks 10 and 500, the red line of the HF list features the most emphasized drop in the comparison sample. That means that there are a few words with a remarkable difference in use with respect to all the other words on the list. Specifically, the words *day*, *people*, *time*, *love*, *life*, *learn*, *flourish*, and *join*, which are the words leading that rank, show a more concentrated frequency than those ranking first in the other lists.

Complementarily, Heaps’ Law analysis (middle of Fig. 1) shows that the use of different words in the HF list increases to the second position when all the words are considered, although it also falls to the fourth place once the stopwords are removed. Generally, these trends are confirmed by analyzing the density of the samples, with and without stopwords. As can be seen, the HF list is in the middle of the selected samples (bottom of Fig. 1).

### Emotions and sentiment words

**Figure 2:**
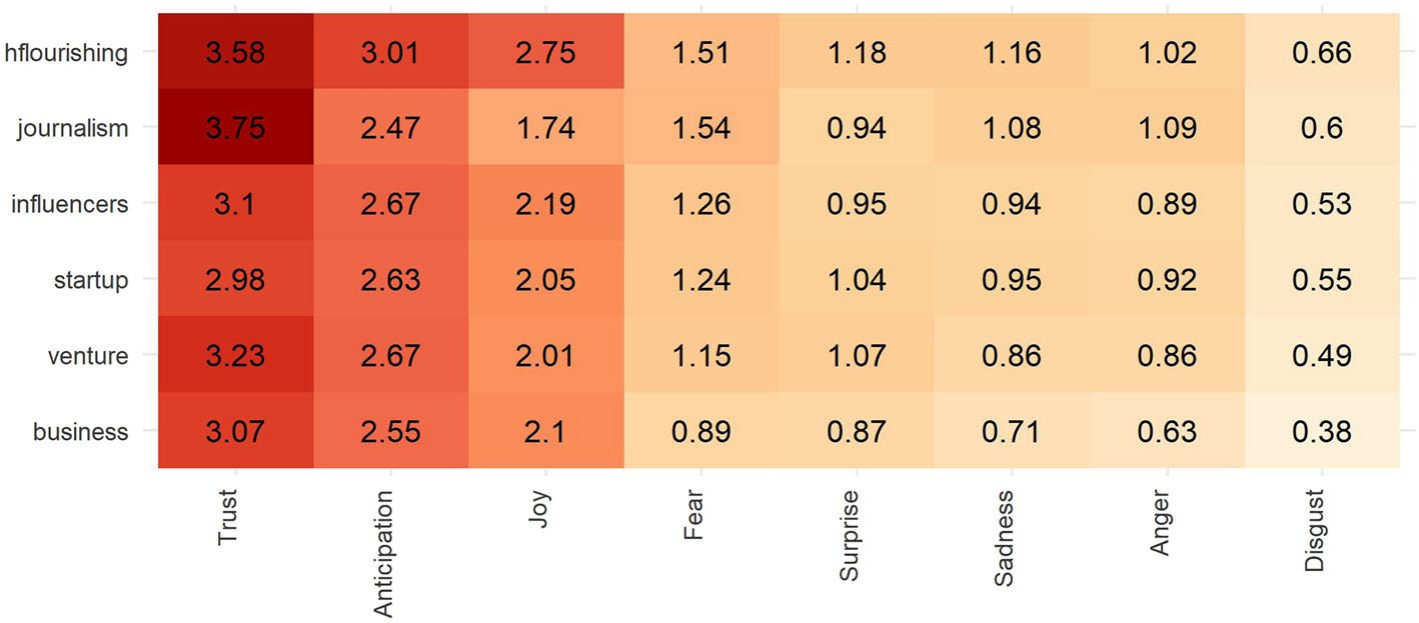
Scores for each of the emotions contained in the NRC lexicon for each selected list. All values were calculated after being normalized with respect to the total number of words in each sample. Graphs were made by the authors, using R programming.

Although the HF list does not show the most extensive vocabulary variety, it does, without a doubt, feature the most significant emotional value, specially for positive emotions. As can be seen in Fig. 2, the journalism list ranks the highest, albeit not by much, in the results for the emotion of trust, while HF stands clearly at the top in emotions such as anticipation, joy, or surprise. Regarding other emotions, all the lists have similar levels, yet it is worth highlighting that the HF list also features emotions of sadness and disgust and ranks second with respect to fear and anger.

**Figure 3:**
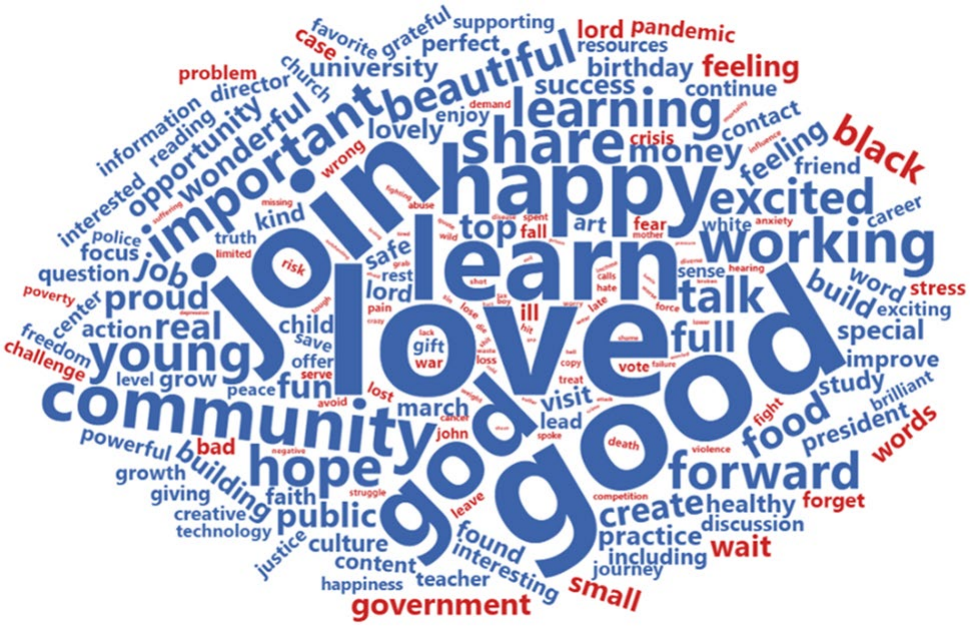
Words from the HF list that contributed most to positive and negative sentiment, shown in blue and red, respectively, and sized proportional to their frequency of occurrence. Some words, such as feeling, are colored in blue and red simultaneously because the repository regards them as holding both sentiment values. Graphic made by the authors, using R programming.

In addition to emotional recognition, a sentiment analysis was conducted to find the specific words in the HF list that contributed the most to a positive or negative sentiment. Figure 3 shows that the positive words of the sample are associated with positive feelings, such as love, good, beautiful, create, excited, or enjoy; there are other positive words related to the act of being together with others, i.e. *join*, *share*, *talk*, *friend*, *culture*, or *contact*; another group is associated with spiritual trends, i.e. *god*, *hope*, *faith*, *lord*, *truth*, or even *safe*; finally, a group of words associated with professional success can be found, i.e. *job*, *success*, *career*, *money*, *university*, *lead*, or *improve*. On the other hand, the words which provided a more negative sentiment to the sample are far fewer, and are associated with personal and even existential troubles that people often find when they are pursuing an achievement, i.e. *wait*, *fear*, *crisis*, *ill*, *problem*, *challenge*, *bad*, *struggle*, *stress*, *anxiety*, or *fall*.

**Figure 4:**
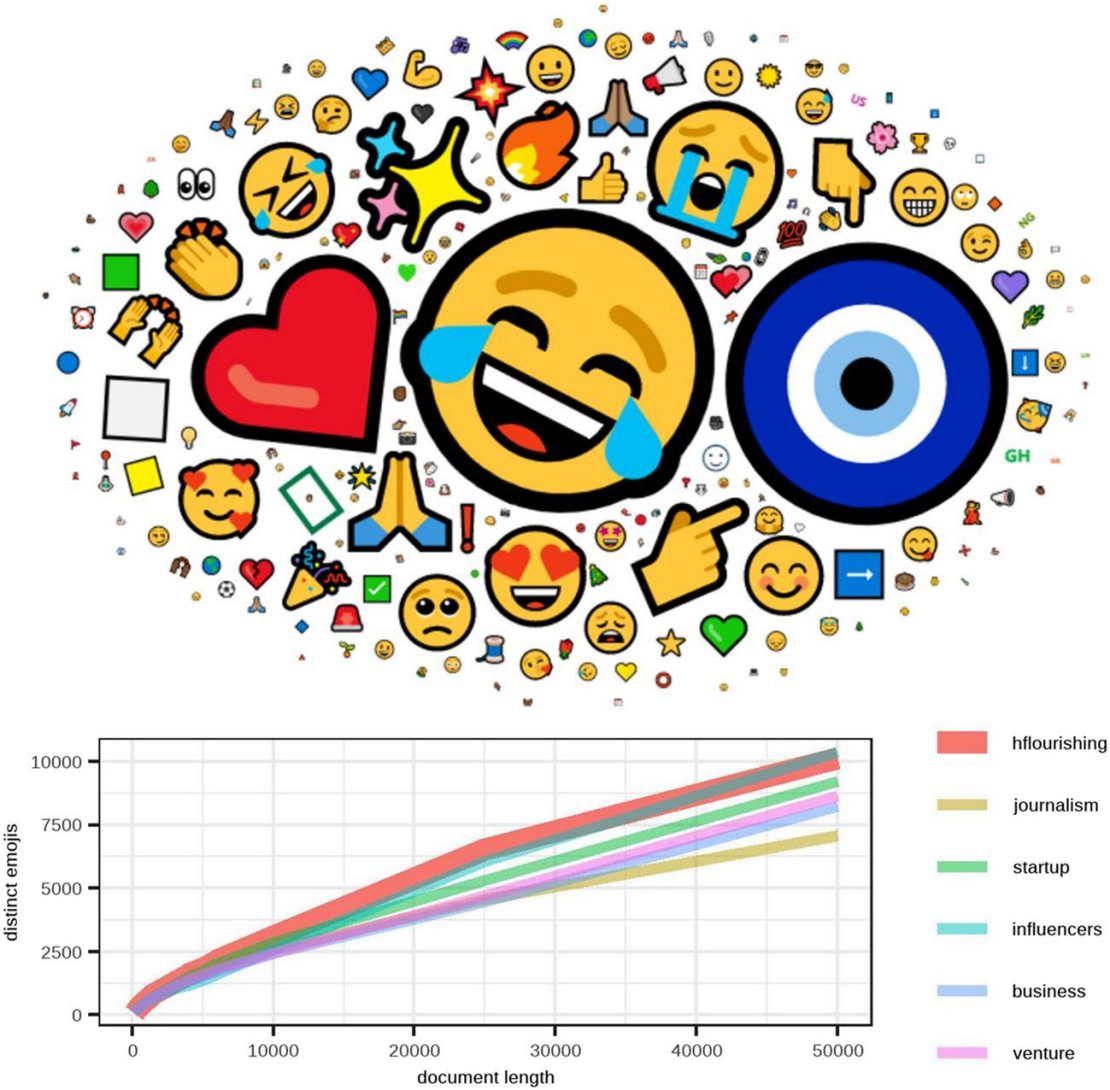
Cloud with the 100 most frequent emojis of the HF list with size proportional to their frequency of appearance. Below Heaps' Law shows the richness of emojis in relation to the length of the document, where the HF list has been highlighted by harder and wider strokes. Graphics were made by the authors, using R programming.

In the analysis of informal expressions of emojis, this study also found a high level of positive emotions. Figure 4 highlights a big face crying with laughter and a large explosive emoji, which implies energy, a very good mood, and trust. These are accompanied by a big red heart, associated with love and passion, and a big Nazar amulet, which implies attention, care, surveillance, and protection. These three emojis are the most frequent in the sample and represent a positive and balanced mood.

In addition to the Nazar amulet, many of the emojis found also express gratitude and signs of respect for cultural variety, i.e. many thank-you signs for different cultures and up to five thank-you emojis with different skin color among the most frequently used 100. Similarly, Fig. 4 shows a number of signs that ask for or give fortune, i.e. the three stars.

Complementing the big red heart, many of the emojis express empathy and the construction of affective and intense relationships; i.e. there are several faces with hearts, smiling, or hugging. In a lower proportion, there are some signs of social commitment and activism, i.e. the loudspeaker, the explosion, or even the index finger pointing downward, which are typically used in social networks when referring to social events and information sharing. On the other hand, the negative emojis are the least, just a medium-small sad face, a small broken heart, and two tiny faces crying.

Along with the analysis of the emojis’ content, Fig. 4 also shows Heaps’ Law applied to the emoji production as compared to the other lists selected. The variety of emoji production is the highest for the first 40,000 words recorded, at which point it moves to second place, only after the influencers list. That shows two things: the fact that the influencers list contains the most relevant Twitter users shows that the high use of emojis is related to social media trends, but also, in the case of HF users, the use of emojis is quite rich and aligned with this trend even though such users have far fewer followers.

### Word types and tenses

**Figure 5:**
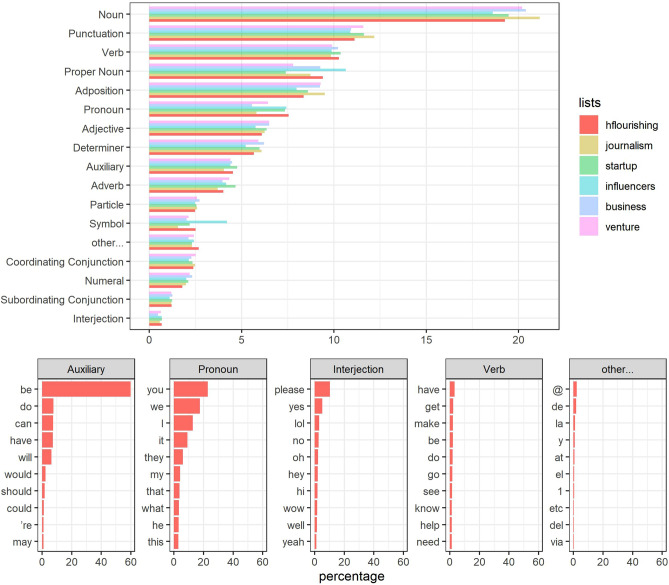
Above: a plot showing the percentages of the different POS tags for each list. Below: those POS tags where the HF list stands out the most, showing which specific words are the most frequent for each of them. Graphs were made by the authors, using R programming.

Regarding entity-recognition analyses using NLP techniques, HF turns out to be the second list in using fewer nouns but almost the first one using verbs (upper part of Fig. 5). This type of vocabulary use can be interpreted as a greater focus on actions rather than on things themselves. The list also features a high number of pronouns as well as auxiliary forms, interjections, and other unrecognized signs returned by *udpipe* package^24^. After a closer analysis of which lemmas are more frequently used in some of these specific POS tags in the HF list (Fig. 5 below), the high frequency of pronouns such as *you*, *we*, or *they* shows the importance of appellative actions and actions done together. Furthermore, the verbs *have*, *get*, *make*, *be*, *do*, *go*, *see*, *know*, *help*, and *need* show a mix of proactive and more cognitive verbs, meanwhile high-frequency interjections like *please*, *yes*, *oh*, *hi*, *wow*, *well*, or *yeah* appeal to a cordial and affirmative treatment. Finally, an inspection of the content of the most frequent POS tags revealed the appearance of words in different languages—particularly Spanish—, pointing out that HF is the list where the presence of foreign words is the highest even though their percentage is still small.

**Figure 6:**
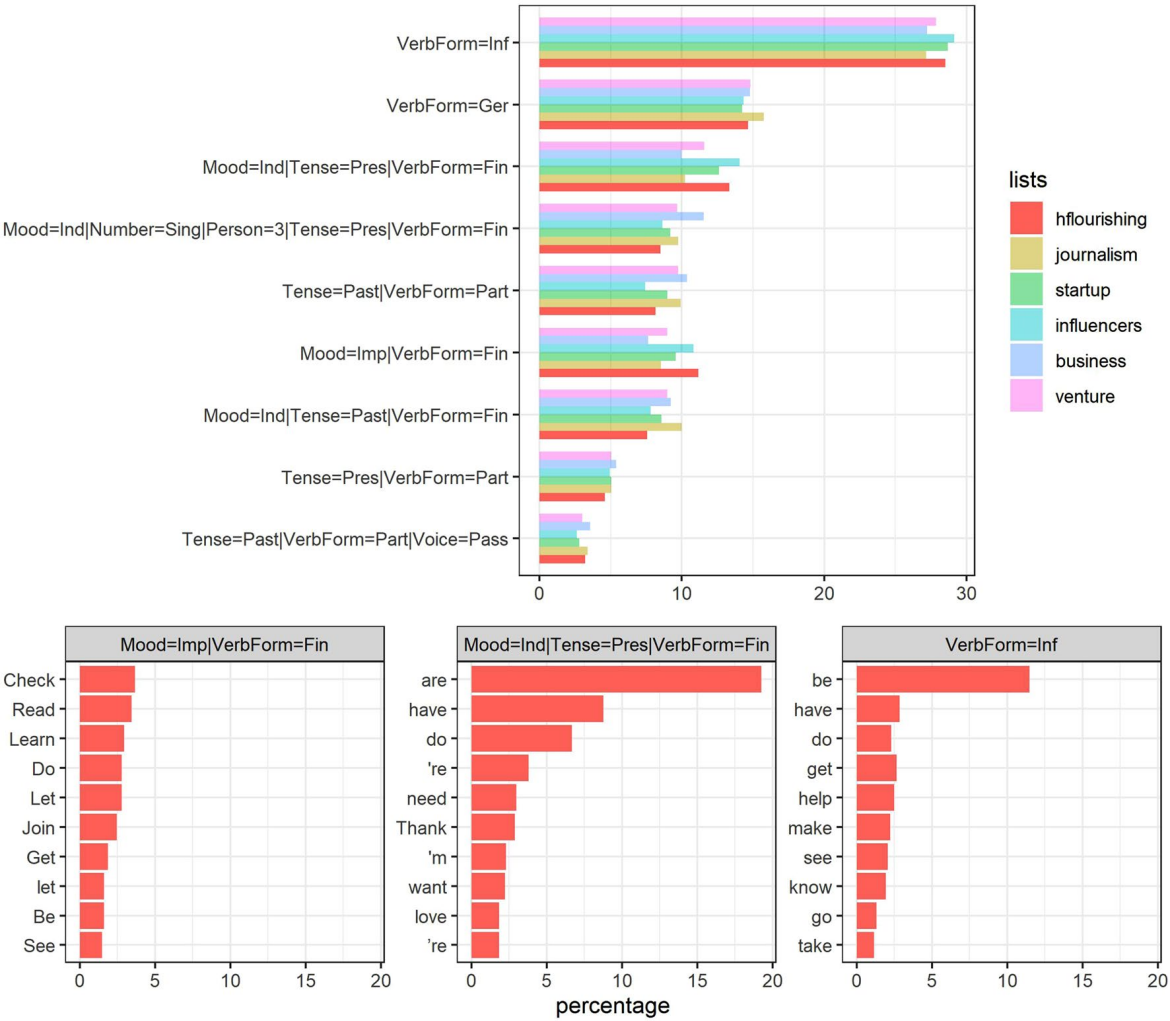
Above: percentages of the different verb tenses for each list. Below: the verb features in which the HF list is most prominent, showing which particular words are most frequent for each. Graphs were made by the authors, using R programming.

Concerning the verb tenses, the HF list highlights the use of the imperative form, the infinitive form, and the present tense (upper part of Fig. 6). The imperative form of a verb is not always a command, but it often conveys a suggestion or proposal being shared by someone. This can be graphically seen by taking a closer look at the words in imperative which occur most frequently in the HF list (bottom of Fig. 6), namely *check, read, learn, do, let,* or *join*. All these are forms of giving and demanding attention and of encouraging people to acquire information and do things together. Regarding the infinitive tense, the use of the verb *to be* stands out, which points to the act of being, in an open and impersonalized way.

### HF topic modeling

**Figure 7:**
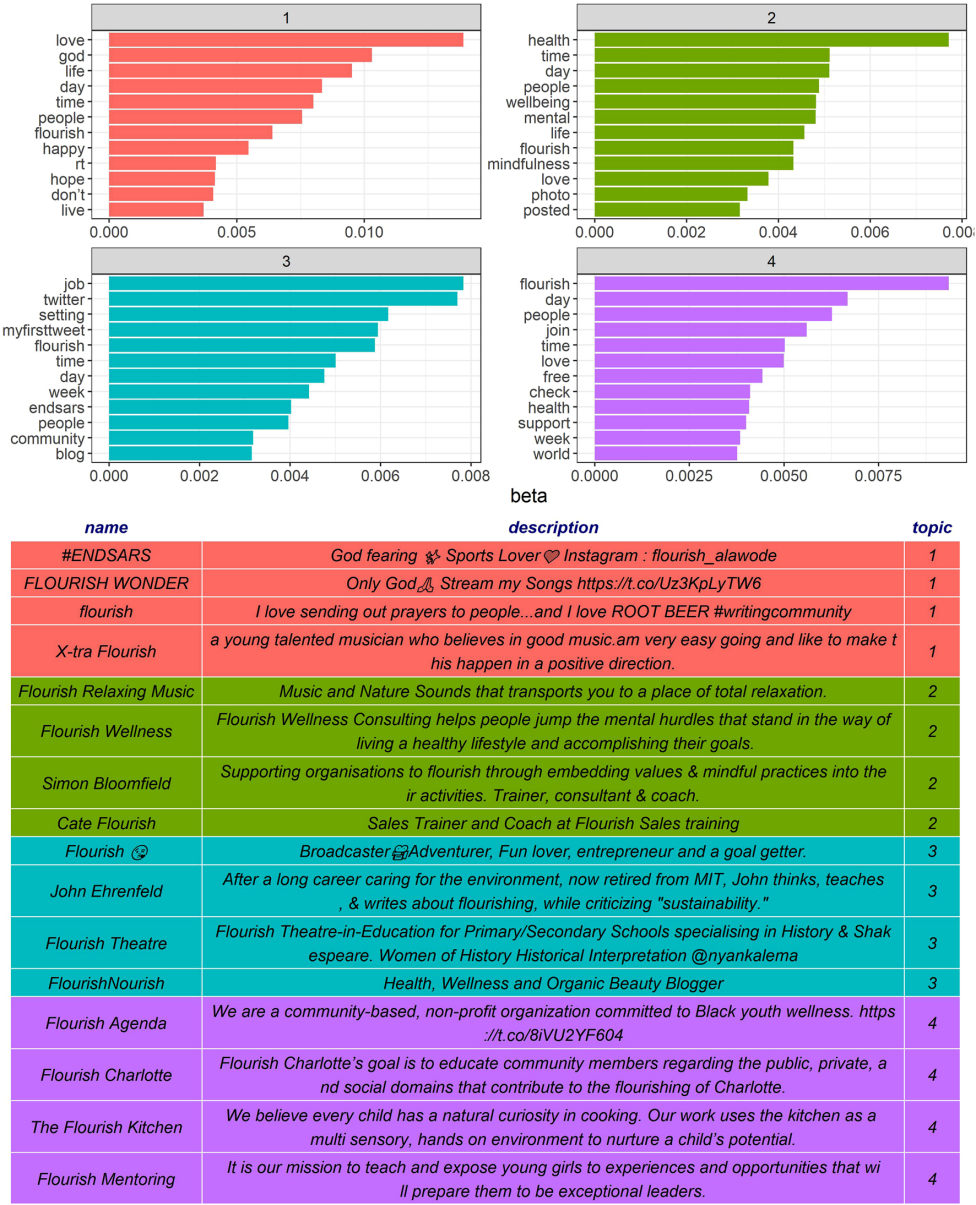
Topic modeling results after applying the LDA algorithm with the k value fixed at 4. The graphs above show the twelve words that relate the most to each topic, while the table below shows the four users—with their user definition—most involved in each topic. The topic numbers do not represent any kind of priority. Graphics were made by the authors, using R programming.

Four main topics were found, characterized mostly by the words *love*, *health*, *job*, and *flourish*. According to the topic modeling techniques, the finding of four different clusters does not mean that they are entirely separate; they can share words that can provide different scores to each topic. That is clearly the case with the HF Twitter list, since Fig. 7 shows how the four MFW of the sample—*day*, *people*, *time*, and *love*—are present throughout all the topics. In continuity with the results found in Fig. 1, it is confirmed the higher frequency of a few content words which are now proven to be highly distributed in the different topics. That is, HF users talk about love, health, work and flourishing transversely, while having a conversation about day, people, time and love.

Looking closer to each topic found, they can be characterized: the first topic led by *love* refers to spiritual feelings, featuring words such as *god*, *hope*, *beautiful*, or *happiness*; the topic led by *health* is about wellbeing and mindfulness; the topic led by *job* refers to Twitter, blogs, and social media activism; and the fourth topic deals more with community, self-care, and caring for the world.

Along with the words that contributed the most to each topic, the LDA algorithm also yielded a gamma value, which connects each topic to users, as seen in the table at the bottom of Fig. 7. In consistency with the previous descriptions: topic one—spirituality—is represented by users associated with music, people who pray to God, and a sports lover; the health topic is represented mainly by therapists, coaches, trainers, and mindfulness organizations; the topic of job is mostly supported by users defined as bloggers, social media activists, and goal getters; finally, the topic of flourishing is represented by users involved in solidary missions, social care, and community development.

## Discussion

Together with the previous objective and quantitative vocabulary analysis, there are other linguistic models more focussed in the performative capacity of a language, opening the discussion about the degree of a vocabulary to connect with the world and with the others.

### Three levels of interaction

The Wellbeing Literacy model (WBL) studies the ability to understand and compose wellbeing messages intentionally interacting at three levels: oneself, others, and the world^3^. Based on the previous findings, there is enough evidence to sustain that the analyzed sample of the HF Twitter list covers these three levels.

Regarding the first level—oneself—most of the profile pictures are self-portraits, which emphasizes the idea of oneself as the subject of the HF processes. Additionally, many of the profile descriptions center on individual activities such as coach, speaker, writer, or author, which specifically establish the self as the object of care and development. Further quantitative evidence was found in the use of pronouns since the pronoun *I* is one of the most frequently used, outweighing the proportional use of pronouns in all the other lists even when ranking third after *you* and *we* in this list. Finally, there were several users who included expressions that are clearly related with one’s own flourishing; for example, *jaydee4all*, who says “God's first love. I FLOURISH”; or *I_will_flourish*, self-described as a “Purpose-Driven Life”.

Nonetheless, the importance of the others in the list is also seen in the high frequency of use identified for the pronoun *you*, and most of the verbs highlighted in Fig. 7 involving a concern with others. Overall, imperative verbs such as *check*, *read*, *learn*, etc. encourage people to practice together, while contributing to enrich the positive sentiment vocabulary in Fig. 4, with terms such as *join, share,* or *create*. Furthermore, it is important not to forget that the second most frequent content word featured is *people*.

The third level of interactions—the world—is widely expressed in many of the most positive words identified in Fig. 4, those implying a certain kind of spiritual need such as *god*, *hope*, *justice*, *freedom*, *culture*, or *art*. Additionally, a general exploration of the profiles revealed many green logos, white backgrounds, or warm colors which belong to entities concerned with global issues: *Arbonne* is described as vegan and cruelty-free; *ScotlandTBP* is a charity oriented to wildlife and people flourishing; *AIDS_Free_World* seeks to avoid inequalities that allow HIV people to flourish; *GreenChristian* states, “Because God made us stewards of HIS planet!”; and *TheFlourishIn* is an initiative where “Flourish is no longer nice to have, it is essential for our species and our planet”. There were also found personal profiles clearly concerned on a world level: *flourish2day* is a business executive that works for a better world and sustainable future; *christianamusk*, places herself “At the nexus of human and planetary flourishing”; and *FlourishGlen* represents a food hall and kitchen “established to benefit both people and planet.”

### Wellbeing literacy capabilities

The WBL project recognizes that a new language is a powerful tool for transformation, involving the recognition and production of new human realities as it entails the following capabilities: (a) knowledge and vocabulary; (b) multimodal comprehension skills; (c) multimodal composition skills; (d) context awareness and adaptability; and (e) intentionality to achieve wellbeing^3^.

The first feature is present in the analyzed tweets through words and basic facts regarding what HF could be. The tweets contained a variety of HF-related synonyms or close words [These searches, and others, can be made directly by the reader using this online resource https://florecimientohumanotec.shinyapps.io/hf_twitter_app/, e.g. the term *wellbeing* -and derivatives such as *well-being*- which is employed in more than 3600 tweets; the term *mental health*, which appears in more than 2700 tweets; the term *happiness,* which is used in almost 2000 tweets; the concept of ethics, which is present in 371 tweets; the term *virtue* appearing in 297 tweets; or the term *eudaimonia,* present in only 24 tweets. Of course, these last three terms are highly academic and less found in more informal and popular media such as Twitter.

Next, the multimodal comprehension and composition skills -second and third mentioned capabilities- can be supported by the different ways in which users identify themselves as associated with HF. For example, several users affirmed to be authors or writers, implying the presence of a writing composition skill; others featured oral composition skills are present in speakers or coaches; others alluded to more cognitive skills, such as students and teachers; yet other users admitted to more physical and sensitive interests such as food, restaurants, and therapists. Finally, there are also those who relate to HF through gardening, decoration, and design composition skills. Regarding the area of multimodal comprehension, the following results can be seen from the produced tweets: almost 6000 employed the word *book*; more than 5000 used *photo*; up to 2400 included the term *music*; more than 1200 used the word *song*; and more than 1300 mentioned *film*.

The context awareness and adaptability aspect is more difficult to assess as it requires a closer reading of the tweets; however, certain clues can be relied on. When compared to the others, the HF list contains a greater number of auxiliary verb forms, as shown in Fig. 5, which points to a high level of grammatical nuances that are not carried out by the verbs or the sentences themselves; this shows an acute awareness of the mode in which the interaction takes place—a conditional, a future, a possibility, or a requirement. Additionally, interjections are a type of vocabulary that reveal interactions, especially those where reactions play an important role in the conversation. Similarly, the high use of terms concerning others and the care for the world featured in the HF list confirm the tweets’ sensitivity towards contextualization.

Finally, intentionality, which is defined as the use of the language to effectively promote wellbeing, constitutes the clearest language capability seen in the analyzed sample. It is first seen in the users’ obvious inclination towards practical activities such as coaching, therapy-giving, or restoration, among others; it is reflected in the importance of pronouns like *you* and *we* in the analysis shown in Fig. 6. Another sign is the constant mention of common activities through verbs like *join* or *share*, as well as the imperative forms, i.e., *check*, *read*, *learn*, and *do* (Fig. 7). All of these convey an idea of HF which goes beyond the self trying to be intentionally incardinated in the world. Even more significant was the presence of many social entities or company profiles, as well as the importance given to topics related to jobs, success, or career, and many others concerned with the planet and social justice.

### Academic vs. social media HF understanding

Without being exhaustive, the current study did notice a differentiation between the use of both words together -human flourishing-, and the single use of the verb flourish as applied to a process of human development and qualification. The term human flourishing was found to be more restricted to academic and scientific profiles, while the use of flourish is much more widespread, with a significant number of users from diverse cultural communities, who use it in more informal contexts. However, the present study suggests that they truly share a common ground^31^, since in Fig. 7 no specific topic was either identified in relation to such a difference or reflected in the users association with the topics.

Previous studies on the academic use of HF have shown that the most relevant academic subject areas addressing the concept turned out to be Social Sciences, Arts and Humanities, Medicine, Psychology, Business, and Environmental Sciences^32^, which largely coincides with the relevance of the topics found in the current Twitter analysis: people and community; love and beauty; health, mental health, and well-being; and work, success, or employment. In addition, in both cases, there are some themes that cross-connect all these topics. In the case of academic production, it is the philosophical approach through keywords such as eudaimonia or virtue^32^; while, in social media, the feeling that users have every day and the empathetic and generous mood they share can be translated as a popular idea of virtue and eudaimonia, which moves from valuing oneself to valuing others encouraging them to act in the world.

## Limitations and further studies

The study is limited to a particular social media platform, namely Twitter, implying two kinds of bias: firstly, there are more social media platforms that could be analyzed as sources for the study of HF; secondly, the study should also take into account the bias that any social media entails on its own, since not everyone has the same kind of access to Internet or even the time to be active in these kinds of environments.

Regarding the emojis analyzed, their meaning was simply interpreted by the authors from the standpoint of common social media readers; however, there are already studies that focus on evaluating the emotional value of emojis, among them^33^, or^34^. These kinds of measures can be added quantitatively to the sentiment and emotional analysis in future studies on HF in social media.

Regarding the sentiment analysis performed, although the Lexicon-Based Methods used have been proven robust, less domain-dependent, and useful for social media^19^, there are also more sophisticated methods using NLP specifically for sentiment analysis that are being tested and which will be able to be applied in this kind of studies^35^.

As for the analyzed database, it is worth mentioning that it is also possible to search for the use of the terms HF on Twitter through the API, i.e., tweets where the terms are used. This study chose to analyze the tweets of users who defined themselves as associated with HF, understanding that the use of the terms in a tweet responds more to an occasional HF statement and not so much to the ongoing conversation of a community. However, other studies could take advantage of this possibility and complete it.

Finally, the analysis of the most frequent words, as well as their grammatical type, position, and temporal form, was deeply developed by the linguistic inquiry and word count method inaugurated by Pennebaker^36^. Although in this work some observations have been made in this sense, it would be possible to carry out a more comprehensive analysis under the Pennebaker approach, making the psychological aspects of the natural language more explicit.

## Conclusions

The term *human flourishing* (HF) is employed by a limited number of users on Twitter; however, there are many more who just use the term *flourish* to refer to a human process of development and care. Although HF is primarily an individual-centered process, many of the profiles found belong to private and social entities that used one or both terms in connection with professional and social initiatives.

Compared to other Twitter lists, HF has an average variety of vocabulary, which can be improved due to the very high frequency of words such as *day*, *people*, *time*, *love*, *life*, *learn*, *flourish* and *join*. Despite this accumulation, a significant variety of topics—from personal and health to social and business—converge on the HF list, having as a constant the way users empathetically share their moods and refer to other people, society and the planet every day.

The HF list also stands out noticeably when emotions are considered, especially positive emotions. Among the compared lists, it is the one with the highest scores for joy, anticipation, and surprise, and the second one regarding trust. This strongly recalls Fredrickson’s proposals^37^ on the importance of positive emotions for HF.

The high presence of emotional scores is supported by a rich use of emojis, most of them positive, encompassing a wide area of expression: some of them are concerned with the self; others express care, support, or sympathy; and yet others are related to different cultures, emphasizing the variety and popularity of approaches.

After examining words that denote positive and negative sentiments, the HF list produced a high level of the first ones and a much lower level of the second ones. The positive words that stand out are those associated with positive feelings, words referring to social acts of being together with others, positive terms from spiritual trends, and many others related to professional success.

The HF list meets most wellbeing literacy model (WBL) requirements: vocabulary, multimodal comprehension and composition, context awareness, and intentionality to achieve wellbeing^3^. This last feature stands out the most because, although the topic is based primarily on subjective and spiritual states and feelings, the analyzed vocabulary reveals quite a high intentionality, encouraging people to carry out actions in the world, both for their own success but also for the sake of others and the world.

In this sense, and at least on the Twitter social media platform, HF is a concept that proves to extend beyond individual wellbeing not only because it tends towards the spiritual realm^38^ but also due to the high levels of social commitment and professional accomplishment that it involves.

## Data Availability

The datasets analysed during the current study are available in the Figshare repository: 10.6084/m9.figshare.20541543.
